# Surgical Management of Congenital Hyperinsulinism in a Resource-Limited Setting

**Published:** 2013-04-01

**Authors:** Santosh B Kurbet, Gowda Parameshwar Prashanth, Vijay C Pujar, Manisha R Bhandankar, Sangappa M Dhaded, Mahantesh V Patil

**Affiliations:** Department of Pediatric Surgery, Jawaharlal Nehru Medical College Belgaum Karnataka, India; 1 Department of Pediatrics, Jawaharlal Nehru Medical College Belgaum Karnataka, India

**Dear Sir**

Persistent hyperinsulinemic hypoglycemia of infancy (PHHI) or congenital hyperinsulinism is considered the most common cause of severe hypoglycemia in the neonatal period with a high risk of subsequent brain damage and seizures, and sometimes mortality [1, 2]. There are no published studies from India reporting the results of surgical management of PHHI. 

A 15-day old female baby was referred to our center for evaluation of intractable hypoglycemic seizures. The baby was full-term, hospital-born via spontaneous vaginal route with birth weight 3.5 kg. There was no history of perinatal asphyxia, jaundice, or maternal diabetes. On examination, there was no hypothermia, dehydration, or clinical signs of sepsis. There were no dysmorphic features. 

Laboratory investigations revealed hemoglobin 14.6 g/dL, total leukocyte count 12X109/L, and platelet count 290X109/L. C-reactive protein (CRP) was negative. During the hospital stay, the infant had repeated generalized and focal clonic seizures during documented hypoglycemic episodes requiring glucose infusion rate (GIR) up to 14 mg/kg/min. There was no ketosis, hyperammonemia, or lactic acidosis. Serum insulin level estimated in a critical sample (blood glucose=22 mg/dL) was 6µL/mL with normal serum cortisol levels. Serum fatty acids and C-peptide levels were not estimated. CT abdomen and MRI brain were normal. A trial of diazoxide for 7 days (up to 20 mg/kg/day) with hydochlorothiazide (10 mg/kg/day) and add-on subcutaneous octreotide (up to 40μg/kg) were ineffective. In view of failure of medical line of management, surgical intervention was performed on day-28. 

Intraoperatively pancreas was examined for nodules; none were found. Pancreas was mobilized from its tail to the head. The pancreas including the uncinate process, part of the head was resected together after dissecting beyond the mesenteric vessels and portal vein. The common bile duct was identified and spared. Histopathology of the resected specimen revealed hypertrophied beta cells with large abnormal nuclei and irregular sized islets of Langerhans throughout the resected pancreas. Following surgery, the infant was maintained on diazoxide and octreotide which were gradually tapered and stopped after 3 weeks. The patient, currently under follow-up is seizure-free and normoglycemic with appropriate dietary measures at nine months follow-up. 

Neonates with PHHI are often macrosomic with a subtle form of facial dysmorphism. [3] In our case, syndromes associated with hyperinsulinism like Beckwith-Wiedemann syndrome, Kabuki syndrome, Costello syndrome, Perlman syndrome, etc., were ruled out as the neonate had no dysmorphic features. Hyperammonemia and certain organic acidemias may be associated with hyperinsulinism. [4] Persistent severe non-acidotic, non-ketotic hypoglycemia with elevated insulin levels and a glycemic response to glucagon confirmed the diagnosis in our case.

The two different histopathological forms of PHHI, focal pancreatic adenomatous hyperplasia and diffuse β-cell abnormality, can be identified by 18F-dopa positron emission tomography (PET) scan. A lesser known third variety (atypical histological form) is described recently. [5] Percutaneous transhepatic pancreatic venous sampling can also identify foci of insulin hyper secretion. Intra-arterial calcium stimulation test is not widely practiced owing to the risk of celiac artery infarction. [1] In our case, preoperative identification of the lesion was not possible due to non-availability of 18F-dopa PET scan in our center; pancreatic venous sampling was not done due to logistic problems. However, histopathological examination of resected pancreatic tissue showed a diffuse hyperplasia of β-cells with large nuclei and abundant cytoplasm justifying near-total pancreatectomy.

Surgery is recommended when medical and dietary therapies fail to achieve euglycemia. In focal form, the lesion is excised. Diffuse variety requires subtotal or near-total (>95%) pancreatectomy. Even diazoxide-responsive patients should undergo further investigations because in case a focal pancreatic lesion is found, surgical excision is potentially curative. Lovvorn et al. reported that near-total pancreatectomy is more likely than subtotal resection to restore euglycemia, irrespective of underlying pathology. [6] Re-look surgery is considered in cases with persistent hypoglycemia requiring high GIR. 

Following subtotal pancreatectomy, up to 70% and 86% children develop permanent diabetes mellitus, respectively, by puberty. [4] PHHI carries a high risk of development of severe mental retardation (44%) and epilepsy (25%). [1, 2] Our patient is seizure-free, without signs of neurological dysfunction at nine months age. 

Favorable long-term prognosis in PHHI necessitates early recognition and intensive monitoring to avoid hypoglycemia. Late referrals, often seen in our set-up, are known to be associated with high rates of brain injury. Advanced imaging technique (PET scan) and rapid genetic analysis can identify surgically curable (focal) forms. [7] Unfortunately, these diagnostic tools are not available in most of the developing-country centers. ‘Blind’ pancreatic resections are usually performed without preoperative localization of the lesion. We suggest early surgery in suburban set-ups to avoid unrecognized hypoglycaemia and resultant brain damage. The present case illustrates the successful surgical management of congenital hyperinsulinism in a resource-limited setting.

## Footnotes

**Source of Support:** Nil

**Conflict of Interest:** None

## References

[1] ( 2000). Aynsley-Green A, Hussain K, Hall J, Saudubray JM, C Nihoul-Fékété C, De Lonlay P, et al. Practical management of hyperinsulinism in infancy.. Arch Dis Child Fetal Neonatal Ed..

[2] ( 2001). Menni F, De Lonlay P, Sevin C, Touati G, Peigné C, Barbier V. Neurologic outcomes of 90 neonates and infants with persistent hyperinsulinemic hypoglycemia.. Pediatr..

[3] ( 2002). De Lonlay P, Cormier-Daire V, Amiel J, Touati G, Goldenberg A, Fournet JC, et al. Facial appearance in persistent hyperinsulinemic hypoglycemia.. Am J Med Genet..

[4] ( 2011). Arnoux JB, Verkarre V, Saint-Martin C, Montravers F, Brassier A, Valayannopoulos V, et al. Congenital hyperinsulinism: current trends in diagnosis and therapy.. Orphanet J Rare Dis..

[5] ( 2008). Hussain K, Flanagan SE, Smith VV, Ashworth M, Day M, Pierro A, et al. An ABCC8 gene mutation and mosaic uniparental isodisomy resulting in atypical diffuse congenital hyperinsulinism.. Diabetes.

[6] ( 1999). Lovvorn HN 3rd, Nance ML, Ferry RJ Jr, Stolte L, Baker L, O'Neill JA Jr, et al. Congenital hyperinsulinism and the surgeon: lessons learned over 35 years.. J Pediatr Surg.

[7] ( 2011). Banerjee I, Skae M, Flanagan SE, Rigby L, Patel L, Didi M, et al. The contribution of rapid KATP channel gene mutation analysis to the clinical management of children with congenital hyperinsulinism.. Eur J Endocrinol.

